# Anticholinergic medication exposure predicts poor physical capability: Findings from a large prospective cohort study in England

**DOI:** 10.1016/j.maturitas.2020.07.006

**Published:** 2020-12

**Authors:** Kaisa R. Yrjana, Victoria L. Keevil, Roy L. Soiza, Robert N. Luben, Nicholas J. Wareham, Kay-Tee Khaw, Phyo K. Myint

**Affiliations:** a Ageing Clinical & Experimental Research (ACER) Team, Institute of Applied Health Sciences, University of Aberdeen, Aberdeen, UK; b Faculty of Health and Medical Sciences, University of Copenhagen, Denmark; c Department of Medicine for the Elderly, Addenbrooke’s Hospital, Cambridge, UK; d Cambridge Institute of Public Health, University of Cambridge, Cambridge, UK; e Department of Medicine, University of Cambridge, Cambridge, UK; f Department of Medicine for the Elderly, Aberdeen Royal Infirmary, Aberdeen, UK; g Department of Public Health & Primary Care University of Cambridge, Cambridge, UK; h Medical Research Council Epidemiology Unit, University of Cambridge, Cambridge, UK

**Keywords:** Physical capability, Anticholinergic burden, Older people

## Abstract

**Objectives:**

To examine whether anticholinergic medication exposure in middle and late life is associated with physical capability.

**Study design:**

We used data from 8477 men and women who had enrolled in the European Prospective Investigation of Cancer-Norfolk study at baseline (1HC; 1993–1997) and who had attended its third health examination (3HC; 2004–2010). Medication history at the 1HC and 3HC was used to score participants according to the Anticholinergic Cognitive Burden (ACB) Scale at baseline and 3HC; participants were categorised as ACB = 0, ACB = 1, ACB>2.

**Main outcome measure:**

At 3HC, physical capability was objectively measured by: usual walking speed, maximum grip strength, timed chair stands speed (TCSS) and standing balance. Linear and logistic regression models examined prospective and cross-sectional associations between ACB and physical capability, controlling for co-morbidity, sociodemographic and lifestyle factors.

**Results:**

The analyses included 3386 men and 4110 women who were 56.4 (SD 7.9) and 55.0 (7.7) years old respectively at baseline and 69.4 (8.1) and 67.9 (8.0) years old at follow-up. Significant cross-sectional and prospective relationships were observed for all physical capability measures in women, except grip strength. For example, women with ACB ≥ 2 compared with ACB = 0 at baseline had 0.07 m/s (95 % CI -0.11, -0.03) slower usual walking speed, 2.61 stands/min (-4.17, -1.05) slower TCSS and higher odds of being unable to complete a tandem stand (odds ratio 2.40, 95 % CI 1.53, 3.76). These trends were observed in men but were less consistent in prospective analyses.

**Conclusion:**

Exposure to anticholinergic medication predicts poor physical capability and is a potentially reversible risk factor.

## Highlights

• Exposure to anticholinergic medication predicts lower physical capability.
• Associations were seen in a sample of men and women aged 48–92 years from a general population.
• Differences in walking speed were equivalent to 3–9 years’ difference in age.
• Exposure to anticholinergic medication is prevalent and potentially modifiable.
• Identifying modifiable risk factors is necessary to optimise late-life function.

## 1 Introduction

Many commonly prescribed medications such as anti-emetics, antihistamines and tricyclic antidepressants are known to have anticholinergic properties. Approximately 20–50 % of older adults have a prescription for at least one such medication and, in the UK, the proportion of older people prescribed anticholinergic medications has steadily increased over the last few years [1,2]. This trend is worrying considering emerging evidence that anticholinergic medication exposure is associated with adverse health outcomes. For example, exposure to anticholinergic medications has been associated with late-life cognitive impairment, higher risk of falls and incident stroke [[3], [4], [5]].

Physical capability refers to an individual’s ability to complete the everyday activities of daily life. Objective measures of physical capability such as grip strength, usual walking speed, timed chair stands speed (TCSS) and standing balance predict mortality, nursing home admission and hospitalisation in older people [6,7]. The association between anticholinergic medication exposure and physical capability has been previously explored [4,[8], [9], [10], [11], [12], [13]] with studies demonstrating a negative relationship. However, not all results have supported an association between anticholinergic medication exposure and lower physical capability, perhaps because studies have focused on older patient groups with a limited age range and some have had small sample sizes. Understanding the relationship between anticholinergic medication use and physical capability is important, since this exposure is prevalent and potentially modifiable. Additionally, preserving physical capability into late-life could improve the functional health of older adults, reducing disability and dependency [14]. Therefore, the aim of the current study is to explore the associations between anticholinergic medication exposure, as measured by the Anticholinergic Cognitive Burden (ACB) Scale [15], and a range of objective measures of physical capability in a large population-based sample of middle-aged and older British men and women.

## 2 Methods

### 2.1 Participants

The participants used in this report were drawn from the European Prospective Investigation of Cancer (EPIC)-Norfolk study, which recruited 25 639 participants between the ages of 39 and 79 years from general practice registers in and around the city of Norwich (Norfolk, England) between 1993 and 1997. These participants attended a baseline health examination (1HC) and the study design and follow-up has been previously described [16]. The present study utilised data from 8623 participants who also attended the 3rd health examination (3HC) of the EPIC-Norfolk study [17]. During the 3HC, men and women who were now aged 48–92 years old attended a central research clinic and completed tests of physical performance and muscle strength. Compared to those who took part in the 3HC, participants who did not return were more likely to be from lower socioeconomic classes, older, smokers and have had higher BMI and blood pressure at baseline [17]. Signed informed consent was obtained from the participants at baseline and renewed at 3HC. Ethical approval was obtained from the Norfolk Local Research Ethics Committee and the East Norfolk and Waveney NHS Research Governance Committee.

### 2.2 Measurements

At the 1HC participants completed a health and lifestyle questionnaire and self-reported their educational status, social class, physical activity [18], smoking status, alcohol consumption, prevalent illnesses and medication history. This assessment was repeated at the 3HC using a similar questionnaire, although educational status and social class were not ascertained again. Additionally, the 3HC health and lifestyle questionnaire did not specifically ask about prevalent illnesses. Therefore, in our analyses prevalent illness at the 3HC was estimated using information combined from health and lifestyle questionnaires administered at the 1HC and 3 years later at the second health examination (2HC 1998–2000). At both time points the prevalent illnesses included in analyses were: myocardial infarction, stroke, cancer, diabetes, asthma, and arthritis. Information on depression requiring treatment was available but was not included as a co-variable in regression analyses due to multicollinearity.

Physiological parameters such as weight, and height were measured by trained research nurses at a research clinic appointment at both the 1HC and 3HC. All measurements were made using standardised equipment following standard operating procedures (SOPs).

Medications listed in the Anticholinergic Cognitive Burden (ACB) scale [15] were cross-matched with the medication histories reported by participants at both the 1HC and 3HC. The cross-matching process identified exact or similar entries with respect to both the generic and brand names of medications. Each medication was then assigned a corresponding ACB scale score. Medications with no anticholinergic properties i.e., no match, were scored 0. Medications with possible anticholinergic effects were given a score of 1 and medications with definite anticholinergic properties were given a score of 2 or 3, depending on potency. The total ACB scale score was calculated as the sum of all scores assigned to medications in each participant’s list at the 1HC and 3HC. Participants were then categorised according to their total ACB scale score at the 1HC and 3HC: ACB = 0, ACB = 1 and ACB ≥ 2.

### 2.3 Outcome measures

Physical capability measures were collected by trained research nurses at a central research clinic during the 3HC. 8477 participants completed the physical capability assessment. Usual walking speed (metres/second [m/s]) was estimated by calculating the average time taken to walk a 4-metre course two times, with walking aids if necessary. Grip strength was measured twice in each hand using a Smedley dynamometer (Scandidact, Kvistgaard, Denmark) and the maximum strength out of all attempts (Kilograms, Kg) was used in analyses. Timed chair stands speed (TCSS, stands/minute) was measured by timing participants as they rose from a chair five times. They were told to perform the task as quickly as possible with their arms folded across their chest and feet flat on the floor. Standing balance was recorded by asking the participants to stand for 10 s with their feet apart in parallel, semi-tandem and tandem positions. As reported previously, very few members of the EPIC-Norfolk cohort were unable to complete the side-by-side and semi-tandem stand [19]. Therefore, standing balance was dichotomized into those who could and those who could not hold the last standing test (tandem stand) for 10 s.

If participants were unable to take part in any of the physical capability tests for health reasons, or because they were unable to perform the task, this was documented.

### 2.4 Statistical analysis

Statistical analyses were performed using SPSS version 24.0 (SPSS Inc., Chicago, IL, USA). Sample characteristics and descriptive statistics were presented at baseline and the 3HC by ACB scale categories. Differences between ACB categories were assessed using the Chi-squared test for categorical variables and analysis of variance (ANOVA) for continuous variables. The associations between ACB categories and grip strength, usual walking speed and TCSS were explored using linear regression models, as data were normally distributed in these variables. Associations between ACB category and standing balance were evaluated using logistic regression.

In supplementary analyses, logistic regression analyses were performed to evaluate associations between ACB categories and poor grip strength, defined as <20 kg for women and <30 kg for men [20] and poor gait speed, defined as <1 m/s for all participants [21]. Additionally, logistic regression was used to evaluate associations between those able versus unable to take part in the TCSS test (n = 794).

All of the analyses were performed separately for men and women due to differences between sexes in physical capabilities. Multivariable regression analyses were first adjusted for age and comorbidities (prevalent MI, stroke, cancer, diabetes, asthma, arthritis), Model A, and then for social class and educational level, Model B. The effect of lifestyle factors was explored individually with the following models: Model C = B + smoking status, Model D = B + alcohol intake, Model E = B + body mass index (BMI) and Model F = B + physical activity. Finally, model G adjusted for model B as well as all lifestyle factors (smoking status, alcohol intake, BMI and physical activity). The analyses were performed prospectively, with predictors measured at baseline and outcomes at 3HC, as well as cross-sectionally with all variables measured at the 3HC. F-Tests and Likelihood Ratio Tests were used to compare models with and without anticholinergic burden and a two-sided P-value <0.05 was considered statistically significant. Multivariable regression results were given as effect size (mean difference, ‘β' or odds ratio, OR) with 95 % Confidence Intervals (95 % CI).

## 3 Results

### 3.1 Characteristics of the EPIC-Norfolk population

3386 men and 4110 women who attended the 3HC, underwent assessment of physical capability and had complete co-variable data were included. The mean age (standard deviation, SD) of men at baseline was 56.4 (7.9) years and 69.4 (8.1) years at follow up. The corresponding values for women were 55.0 (7.7) years and 67.9 (8.0) years, respectively.

Table 1 demonstrates the characteristics of the participants and crude outcome values by ACB categories at the 3HC. Participants in the higher ACB categories were older, had lower level of educational attainment, higher BMI and increased prevalence of myocardial infarction, stroke and arthritis. Higher ACB category was also associated with being physically inactive and lower physical capability. Similar trends were observed between ACB categories and co-variables measured at baseline and between ACB categories at baseline and physical capability measured over a decade later (Supplementary Data, Table 1).

**Table 1:** Characteristics of the 7496 men and women at the 3HC.

	Men (n = 3386)			Women (n = 4110)		
Characteristic, mean (SD)	ACB = 0 (n = 2556)	ACB = 1 (n = 531)	ACB>2 (n = 299)	ACB = 0 (n = 3064)	ACB = 1 (n = 577)	ACB>2 (n = 469)
**Age, years**	68.4 (8.0)	72.0 (7.4)	69.4 (8.1)*	67.1 (8.0)	72.0 (7.4)	73.4 (7.6)*
**BMI, kg/m^2^**	26.8 (3.4)	27.8 (3.9)	27.7 (3.7)*	26.1 (4.5)	27.3 (5.0)	28.1 (5.0)*
**MI, n (%)**	68 (2.7)	61 (11.5)	46 (15.4)*	24 (0.8)	26 (4.5)	25 (5.3)*
**Stroke, n (%)**	46 (1.8)	31 (5.8)	24 (8.0)*	29 (0.9)	11 (1.9)	18 (3.8)*
**Cancer, n (%)**	168 (6.6)	42 (7.9)	39 (13.0)*	327 (10.7)	69 (12.0)	49 (10.4)
**Diabetes, n (%)**	76 (3.0)	31 (5.8)	22 (7.4)*	51 (1.7)	26 (4.5)	16 (3.4)*
**Asthma (%)**	261 (10.2)	60 (11.3)	40 (13.4)	362 (11.8)	82 (14.2)	86 (18.3)*
**Arthritis, n (%)**	727 (28.4)	181 (34.1)	133 (44.5)*	1140 (37.2)	292 (50.6)	260 (55.4)*
**Depression, n (%)**	301 (11.8)	98 (18.5)	71 (23.7)	776 (25.3)	172 (29.8)	199 (42.4)*
**Social class, n (%)**						
**Professional**	259 (10.1)	50 (9.4)	23 (7.7)	276 (9.0)	42 (7.3)	33 (7.0)
**Manager**	1114 (43.6)	224 (42.2)	132 (44.1)	1250 (40.8)	208 (36.0)	201 (42.9)
**Skilled non-manager**	319 (12.5)	56 (10.5)	42 (14.0)	566 (18.5)	119 (20.6)	84 (17.9)
**Skilled manual**	561 (21.9)	116 (21.8)	67 (22.4)	581 (19.0)	124 (21.5)	71 (15.1)
**Semi-skilled**	255 (10.0)	71 (13.4)	31 (10.4)	330 (10.8)	59 (10.2)	63 (13.4)
**Unskilled**	48 (1.9)	14 (2.6)	4 (1.3)	61 (2.0)	25 (4.3)	17 (3.6)*
**Education level, n (%)**						
**No qualifications**	520 (20.3)	147 (27.7)	71 (23.7)	812 (26.5)	206 (35.7)	166 (35.4)
**O level**	271 (10.6)	44 (8.3)	24 (8.0)	418 (13.6)	70 (12.1)	64 (13.6)
**A level**	1209 (47.3)	255 (48.0)	150 (50.2)	1304 (42.6)	222 (38.5)	181 (38.6)
**Higher degree**	556 (21)	85 (16.0)	54 (18.1)*	530 (17.3)*	79 (13.7)	58 (12.4)*
**Smoking status, n (%)**						
**Current**	110 (4.3)	16 (3.0)	11 (3.7)	131 (4.3)	37 (6.4)	18 (3.8)
**Ex-smoker**	1413 (55.3)	347 (65.3)	197 (65.9)	1084 (35.4)	229 (39.7)	193 (41.2)
**Never smoker**	1033 (40.4)	168 (31.6)	91 (30.4)*	1849 (60.3)	311 (53.9)	258 (55.0)^#^
**Alcohol use, units/week**	8.8 (10.3)	7.0 (7.9)	7.7 (9.6)*	4.5 (5.8)	3.4 (4.9)	3.7 (5.8)*
**Physical activity, n (%)**						
**Inactive**	860 (33.6)	238 (44.8)	154 (51.5)	1006 (33.2)	265 (45.9)	224 (47.8)
**Moderately inactive**	642 (25.1)	138 (26.0)	76 (25.4)	1017 (33.2)	188 (32.6)	124 (26.4)
**Moderately active**	519 (20.3)	88 (16.6)	41 (13.7)	559 (18.2)	76 (13.2)	74 (15.8)
**Active**	535 (20.9)	67 (12.6)	28 (9.4)*	482 (15.7)	48 (8.3)	47 (10.0)*
**UWS, m/s**	1.2 (0.2)	1.1 (0.2)	1.0 (0.3)*	1.1 (0.2)	1.0 (0.3)	1.0 (0.3)*
**Grip strength, Kg**	40.0 (8.2)	37.4 (7.9)	36.1 (7.7)*	24.9 (5.3)	23.4 (5.4)	22.9 (5.9)*
**TCSS, stands/min**	27.5 (8.4)	25.4 (7.9)	23.5 (7.3)*	26.7 (8.7)	24.2 (7.6)	24.1 (7.7)*
**Standing balance, n able (%)**	2208 (94.1)	410 (90.5)	199 (84.7)*	2738 (89.4)	475 (82.3)	349 (74.4)*

*P < 0.001; ^#^P<0.05; BMI: Body mass index; MI: myocardial infarction; UWS: usual walking speed; TCSS: Timed Chair Stands Speed.

The following sections explore associations between ACB categories and physical capability using exposure and co-variable data both from the 1HC (longitudinal analyses) and the 3HC (cross-sectional analyses).

### 3.2 Usual walking speed

In cross-sectional analyses, higher ACB category was independently associated with slower usual walking speed and the association exhibited a dose-response relationship in both men and women (Table 2). For example, men with an ACB score of 1 and >2 had a 0.03 m/s (95 % CI -0.05, -0.01) and 0.07 m/s (95 % CI -0.10, -0.01) slower usual walking speed respectively, compared to men who took no anticholinergic medication. Associations persisted in prospective analyses although were weaker in men. The odds of participants having poor usual walking speed (<1 m/s) by ACB category are shown in Supplementary Data, Table 2. In cross-sectional analyses, participants with higher ACB scale scores had higher odds of slow usual walking speed (Men: ACB = 1, OR 1.43, 95 % CI 1.1, 1.78; ACB>2, OR 1.60, 95 % CI 1.22, 2.11; Women: ACB = 1, OR 1.29, 95 % CI 1.05, 1.58; ACB ≥ 2 OR 1.78 95 % CI1.43, 2.22). In prospective analyses, women with higher ACB scale scores at baseline were more likely to have slow usual walking speed at the 3HC (ACB = 1: OR 1.46, 95 % CI 1.07, 2.00; ACB ≥ 2: OR 2.15, 95 % CI 1.47, 3.14), while no significant association between ACB category at baseline and odds of poor usual walking speed were observed in men.

**Table 2:** Usual walking speed by ACB category in men and women at the third health examination (3HC) and first health examination (1CH).

	Regression Coefficient (95 % Confidence Interval)						
	Model A	Model B	Model C	Model D	Model E	Model F	Model G
**MEN (n = 3386)**							
**3HC**							
ACB = 0	0	0	0	0	0	0	0
ACB = 1	−0.05 (-0.07,0.03)	−0.05 (-0.07, -0.02)	−0.04 (-0.07, -0.02)	−0.04 (-0.07, -0.02)	−0.04 (-0.06, -0.01)	−0.04 (-0.06, -0.02)	−0.03 (-0.05, -0.01)
ACB ≥ 2	−0.08 (-0.11, -0.05)*	−0.08 (-0.11, -0.011)*	−0.08 (-0.11, -0.05)*	−0.08 (-0.11, -0.05)*	−0.07 (-0.10, -0.05)*	−0.07 (-0.10, -0.05)*	−0.07 (-0.10, -0.01)*
**1HC**							
ACB = 0	0	0	0	0	0	0	0
ACB = 1	−0.04 (-0.07, -0.01)	−0.04 (-0.07, -0.01)	−0.04 (-0.07, -0.01)	−0.04 (-0.07, -0.01)	−0.03 (-0.07, -0.01)	−0.04 (-0.07, -0.01)	−0.03 (-0.05, -0.01)
ACB ≥ 2	−0.03 (-0.08, 0.02)^#^	−0.03 (-0.08, 0.02)^#^	−0.03 (-0.07, 0.03)^#^	−0.03 (-0.07, 0.02)^#^	−0.02 (-0.07, 0.03)	−0.03 (-0.07, 0.02)	−0.03 (-0.07, 0.03)
**WOMEN (n = 4110)**							
**3HC**							
ACB = 0	0	0	0	0	0	0	0
ACB = 1	−0.05 (-0.07, -0.03)	−0.05 (-0.07, -0.03)	−0.05 (-0.07, -0.03)	−0.05 (-0.07, -0.03)	−0.04 (-0.06, -0.02)	−0.04 (-0.07, -0.02)	−0.03 (-0.05, -0.01)
ACB ≥ 2	−0.11 (-0.13, -0.09)*	−0.11 (-0.13, -0.08)*	−0.11 (-0.13, -0.08)*	−0.11 (-0.13, -0.08)*	−0.10 (-0.11, -0.07)*	−0.10 (-0.12, -0.08)*	−0.09 (-0.11, -0.06)*
**1HC**							
ACB = 0	0	0	0	0	0	0	0
ACB = 1	−0.07 (-0.10, -0.04)	−0.07 (-0.13, -0.05)	−0.07 (-0.10, -0.04)	−0.07 (-0.10, -0.04)	−0.06 (-0.10, -0.03)	−0.07 (-0.10, -0.03)	−0.05 (-0.09, -0.02)
ACB ≥ 2	−0.10 (-0.14, -0.06)*	−0.09 (-0.13, -0.05)*	−0.09 (-0.13, -0.05)*	−0.09 (-0.13, -0.05)*	−0.08 (-0.18, -0.04)*	−0.09 (-0.13, -0.05)*	−0.07 (-0.11, -0.03)*

* = p < 0.001 **=p<0.01 ^#^= p < 0.05 (F Test or Likelihood Ratio Test used to compare models with and without ACB). Regression coefficients represent the differences in usual walking speed (m/s) between ACB = 1 or ACB = 2 and ACB = 0 (reference category). 1HC: first health examination; 3HC: third health examination. Model A: adjusted for age and comorbidities (MI, stroke, cancer, diabetes, asthma, arthritis), Model B: adjusted for A and environmental factors (social class, educational level) Model C: B + smoking, Model D: B + alcohol, Model E: B + body mass index (BMI), Model F: B + physical activity (PA), Model G: B + smoking, alcohol intake, BMI and PA (the fully adjusted model).

### 3.3 Grip strength

Higher ACB category was associated with weaker grip strength in a dose-response manner, but only in cross-sectional analyses (Table 3). For example, ACB ≥ 2 was associated with a 1.16 kg weaker grip strength (95 % CI -2.02, -0.30) in men and 0.96 kg weaker grip strength in women (95 % CI -1.44, -0.47). No prospective associations between ACB category and grip strength were observed. The odds of participants having poor grip strength according to ACB category were also assessed (Supplementary Table 3). No strong or consistent associations were found and the only cross-sectional associations in women were significant..

**Table 3:** Maximum grip strength (kg) by ACB category in men and women at the third health examination (3HC) and first health examination (1CH).

	Regression Coefficient (95 % Confidence Interval)						
	Model A	Model B	Model C	Model D	Model E	Model F	Model G
**Men (n = 3386)**							
**3HC**							
ACB = 0	0	0	0	0	0	0	0
ACB = 1	−0.64 (-1.31, 0.03)	−0.65 (-1.32, 0.02)	−0.67 (-1.34, 0)	−0.62 (-1.29, 0.05)	−0.92 (-1.59, -0.26)	−0.59 (-1.26, -0.18)	−0.85 (-1.52, -0.18)
ACB ≥ 2	−1.05 (-1.91, -0.19)**	−1.04 (-1.9, -0.17)**	−1.05 (-1.91, -0.19)**	−1.03 (-1.90, -0.17)**	−1.27 (-2.12, -0.41)*	−0.95 (-1.81, -0.53)**	−1.16 (-2.02, -0.30)**
**1HC**							
ACB = 0	0	0	0	0	0	0	0
ACB = 1	−0.10 (-1.05, 0.86)	−0.10 (-1.06, 0.85)	−0.14 (-1.10, 0.81)	−0.11 (-1.06, 0.85)	−0.32 (-1.27, 0.63)	−0.08 (-1.03, 0.88)	−0.31 (-1.26, 0.64)
ACB ≥ 2	0.44 (-1.12, 2.01)	0.35 (-1.22, 1.91)	0.39 (-1.18. 1.91)	0.35 (-1.22, 1.91)	0.22 (-1.34, 1.77)	0.34 (-1.23, 1.90)	0.70 (-1.30, 1.80)
**WOMEN (n = 4110)**							
**3HC**							
ACB = 0	0	0	0	0	0	0	0
ACB = 1	−0.41 (-0.85, 0.03)	−0.35 (-0.79, 0.09)	−0.39 (-0.83, 0.06)	−0.34 (-0.78, 0.10)	−0.46 (-0.90, -0.01)	−0.28 (-0.72, 0.17)	−0.42 (-0.86, 0.03)
ACB ≥ 2	−0.85 (-1.33, -0.36)**	−0.81 (-1.29, -0.33)*	−0.84 (-1.32, -0.35)*	−0.81 (-1.29, -0.32)**	−0.99 (-1.48, -0.51)*	−0.75 (-1.24, -0.27)**	−0.96 (-1.44, -0.47)*
**1HC**							
ACB = 0	0	0	0	0	0	0	0
ACB = 1	−0.43 (-1.14, 0.27)	−0.43 (-1.13, 0.27)	−0.45 (-1.15, 0.25)	−0.42 (-1.12, 0.28)	−0.52 (-1.44, 0.15)	−0.35 (-1.05, 0.35)	−0.45 (-1.15, 0.25)
ACB ≥ 2	−0.41 (-0.85, 0.03)	−0.35 (-0.79, 0.09)	−0.39 (-0.83, 0.06)	−0.34 (-0.78, 0.10)	−0.41 (-0.94, 0.35)	−0.27 (-1.13, 0.59)	−0.34 (-1.20, 0.51)

* = p < 0.001 **=p<0.01 ^#^= p < 0.05 (F Test or Likelihood Ratio Test used to compare models with and without ACB). Regression coefficients represent the differences in maximum grip strength (kg) between ACB = 1 or ACB = 2 and ACB = 0 (reference category). 1HC: first health examination; 3HC: third health examination. Model A: adjusted for age and comorbidities (MI, stroke, cancer, diabetes, asthma, arthritis), Model B: adjusted for A and environmental factors (social class, educational level) Model C: B + smoking, Model D: B + alcohol, Model E: B + body mass index (BMI), Model F: B + physical activity (PA), Model G: B + smoking, alcohol intake, BMI and PA (the fully adjusted model).

### 3.4 Timed chair stand speed (TCSS)

Higher ACB category was associated with slower TCSS in the 6702 participants who completed this test (Table 4). Cross-sectionally, ACB ≥ 2 was associated with 1.79 stands/min (95 % CI -2.84,-072) and 0.96 stand/min (95 % CI 1.85, -0.08) slower TCSS for men and women respectively compared to those who had an ACB scale score of zero. In prospective analyses, women in the ACB ≥ 2 group had on average 2.61 stands/min (95 % CI -4.17, -1.05) slower TCSS compared to those in the ACB = 0 category, while men with ACB = 1 had 1.58 stands/min (95 % CI -2.74, -0.43) slower TCSS compared to those in the ACB = 0 category.

**Table 4:** Timed chair stands speed by ACB category in men and women at the third health examination (3HC) and first health examination (1CH).

	Regression Coefficient (95 % Confidence Interval)						
	Model A	Model B	Model C	Model D	Model E	Model F	Model G
**Men (n = 3035)**							
**3HC**							
ACB = 0	0	0	0	0	0	0	0
ACB = 1	−0.76 (-1.56, 0.39)	−0.69 (-1.49, 0.11)	−0.70 (-1.50, 0.1)	−0.66 (-1.46, 0.14)	−0.51 (-1.31, 0.29)	−0.54 (-1.34, 0.26)	−0.35 (-1.15, 0.45)
ACB ≥ 2	−2.13 (-3.19,-1.06)*	−2.13 (-3.19, -1.06)*	−2.13 (-3.20, -1.07)*	−2.11 (-3.17, -1.04)*	−1.98 (-3.04, -0.91)*	−1.92 (-2.98, -0.86)*	−1.79 (-2.84, -0.72)*
**1HC**							
ACB = 0	0	0	0	0	0	0	0
ACB = 1	−1.81 (-2.97, -0.66)	−1.81 (-2.96, -0.66)	−1.81 (-2.97, -0.66)	−1.82 (-2.97, -0.67)	−1.66 (-2.81, -0.03)	−1.70 (-2.85, -0.55)	−1.58 (-2.74, -0.43)
ACB ≥ 2	−2.16 (-4.05, -0.26)**	−2.03 (-3.93, -0.14)**	−2.03 (-3.92, -0.13)**	−2.02 (-3.92, -0.13)**	−1.92 (-3.81, -0.03)**	−1.95 (-3.84, -0.05)**	−1.83 (-3.72, 0.06)^#^
**WOMEN (n = 3667)**							
**3HC**							
ACB = 0	0	0	0	0	0	0	0
ACB = 1	−1.41 (-2.18, -0.63)	−1.32 (-2.10, -0.55)	−1,33 (-2.10, -0.55)	−1.28 (-2.06, -0.51)	−1.18 (-1.95, -0.40)	−1.22 (-2.00, -0.44)	−1.06 (-1.83, -0.29)
ACB ≥ 2	−1.36 (-2.24, -0.47)*	−1.33 (-2.22, -0.44)*	−1.32 (-2.21, -0.43)*	−1.31 (-2.19, -0.42)*	−1.06 (-1.95, -0.18)*	−1.23 (-2.11, -0.34)*	−0.96 (-1.85, -0.08)*
**1HC**							
ACB = 0	0	0	0	0	0	0	0
ACB = 1	−0.42 (-1.73, 0.89)	−0.41 (-1.72, 0.90)	−0.41 (-1.72, 0.90)	−0.40 (-1.70, 0.91)	−0.24 (-1.54, 1.07)	−0.31 (-1.61, 1.00)	−0.14 (-1.45, 1.17)
ACB ≥ 2	−2.93 (-4.50, -1.36)*	−2.76 (-4.33, -1.20)*	−2.76 (-4.33, -1.20)*	−2.75 (-4.32, -1.19)*	−2.66 (-4.22, -1.09)*	−2.72 (-4.29, -1.16)*	−2.61 (-4.17, -1.05)*

* = p < 0.001 **=p<0.01 ^#^= p < 0.05 (F Test or Likelihood Ratio Test used to compare models with and without ACB). Regression coefficients represent the differences in timed chair stands speed (stands/min) between ACB = 1 or ACB = 2 and ACB = 0 (reference category). 1HC: first health examination; 3HC: third health examination. Model A: adjusted for age and comorbidities (MI, stroke, cancer, diabetes, asthma, arthritis), Model B: adjusted for A and environmental factors (social class, educational level) Model C: B + smoking, Model D: B + alcohol, Model E: B + body mass index (BMI), Model F: B + physical activity (PA), Model G: B + smoking, alcohol intake, BMI and PA (the fully adjusted model).

Higher ACB category was also associated with inability to participate in the TCSS test due to poor functional ability or health reasons (Supplementary Data, Table 4). In men, significant associations were only observed in cross-sectional analyses, whilst both cross-sectional and prospective associations were observed in women. For example, baseline ACB category was independently associated with higher odds of women being unable to participate in the TCSS test at the 3HC (ACB = 1 OR 1.93, 95 % CI 1.34, 2.77; ACB ≥ 2 OR 1.81, 95 % CI 1.13, 2.89).

### 3.5 Standing balance

ACB category was associated with poor standing balance in both cross-sectional and prospective analyses for women (Table 5). The association also exhibited a dose-response relationship. For example, baseline ACB category was associated with higher odds of being unable to perform a tandem stand (ACB = 1 OR of 1.69, 95 % CI 1.17, 2.42; ACB ≥ 2 OR of 2.40, 95 % CI 1.53, 3.76). For men, associations did not persist in prospective analyses (Table 5).

**Table 5:** Odds of participants being unable to hold tandem stand for 10 s by ACB scale category at the third health examination (3HC) and first health examination (1CH).

	Odds ratios (95 % Confidence Interval)#						
	Model A	Model B	Model C	Model D	Model E	Model F	Model G
**Men (n = 3386)**							
**3HC**							
ACB = 0	1.00	1.00	1.00	1.00	1.00	1.00	1.00
ACB = 1	1.26 (0.93, 1.72)	1.27 (0.93, 1.72)	1.26 (0.93, 1.72)	1.27 (0.93, 1.72)	1.17 (0.85, 1.60)	1.24 (0.91, 1.70)	1.15 (0.84, 1.58)
ACB ≥ 2	1.98 (1.41, 2.77)*	2.00 (1.42, 2.81)*	2.00 (1.42, 2.80)*	2.00 (1.42, 2.80)*	1.90 (1.35, 2.67)*	1.95 (1.38, 2.74)*	1.85 (1.31, 2.61)*
**1HC**							
ACB = 0	1.00	1.00	1.00	1.00	1.00	1.00	1.00
ACB = 1	1.33 (0.91, 1.97)	1.32 (0.90, 1.95)	1.32 (0.90, 1.96)	1.32 (0.90, 1.96)	1.27 (0.86, 1.87	1.29 (0.87, 1.91)	1.30 (0.86, 1.96)
ACB ≥ 2	1.39 (0.71, 2.69)	1.35 (0.69, 2.63)	1.35 (0.69, 2.63)	1.35 (0.69, 2.63)	1.32 (0.68, 2.58)	1.34 (0.69, 2.62)	1.18 (0.57, 2.45)
**Women (n = 4110)**							
**3HC**							
ACB = 0	1.00	1.00	1.00	1.00	1.00	1.00	1.00
ACB = 1	1.29 (1.0, 1.68)	1.24 (0.95, 1.61)	1.23 (0.94, 1.60)	1.24 (0.95, 1.61)	1.16 (0.89, 1.51)	1.20 (0.92, 1.57)	1.13 (0.86, 1.47)
ACB ≥ 2	2.20 (1.70, 2.84)*	2.14 (1.65, 2.77)*	2.14 (1.65, 2.77)*	2.14 (1.65, 2.78)*	1.94 (1.49, 2.52)*	2.08 (1.60, 2.70)*	1.90 (1.46, 2.48)*
**1HC**							
ACB = 0	1.00	1.00	1.00	1.00	1.00	1.00	1.00
ACB = 1	1.75 (1.23, 2.49)	1.79 (1.26, 2.53)	1.79 (1.26, 2.54)	1.79 (1.26, 2.53)	1.68 (1.18, 2.39)	1.74 (1.22, 2.47)	1.69 (1.17, 2.42)
ACB ≥ 2	2.78 (1.83, 4.21)*	2.61 (1.71, 4.0)*	2.58 (1.69, 3.93)*	2.61 (1.72, 4.0)*	2.49 (1.64, 3.81)*	2.56 (1.68, 3.88)*	2.40 (1.53, 3.76)*

* = p < 0.001 **=p<0.01 ^#^= p < 0.05 (F Test or Likelihood Ratio Test used to compare models with and without ACB). Odds ratios represent the odds of being unable to hold a tandem stand for 10 s in ACB = 1 or ACB = 2 categories compared to the reference category, ACB = 0. 1HC: first health examination; 3HC: third health examination. Model A: adjusted for age and comorbidities (MI, stroke, cancer, diabetes, asthma, arthritis), Model B: adjusted for A and environmental factors (social class, educational level) Model C: B + smoking, Model D: B + alcohol, Model E: B + body mass index (BMI), Model F: B + physical activity (PA), Model G: B + smoking, alcohol intake, BMI and PA (the fully adjusted model).

## 4 Discussion

To our knowledge this is the first study to investigate the association between anticholinergic medication exposure and physical capability in a cohort of men and women spanning a wide age-range, originally sampled from the general population. Our results demonstrate that anticholinergic burden was significantly associated with poorer usual walking speed, standing balance and performance in the TCSS test in both cross-sectional and prospective analyses, with evidence of a dose-response association. In prospective analyses associations were stronger and more consistent in women and, in either sex, ACB category was only associated with low grip strength in cross-sectional analyses.

A clinically meaningful change in usual walking speed has been estimated at around 0.5 m/s with differences of 0.10 m/s associated with significant differences in mortality risk [22,23]. Our results were comparable to this magnitude, especially in women, and the differences observed in walking speed across ACB categories were equivalent to between 3 and 9 years difference in age [19]. To the best of our knowledge, other physical capability measures investigated in this study do not have well-defined clinically relevant change values but associations between lower physical capability and higher mortality have been described with a 1 kg difference in grip strength [24] and being unable to hold a tandem stand [25]. Additionally, low grip strength and slow walking speed, as defined in our supplementary analyses, are associated with higher risk of adverse health outcomes [20,21]. Therefore, the differences in physical capability, and odds of poor physical capability, observed across ACB categories were meaningful.

Physical capability is also a meaningful outcome to study. Mid and late-life physical capability predicts important health outcomes such as mortality, institutionalisation, and the onset of disability [24,25]. Physical capability has also been suggested as a possible mediating factor in relationships between anticholinergic medication exposure and health outcomes such as falls [4]. Therefore, identifying factors that are associated with lower physical capability is important since this will inform development of future interventions targeted at improving health in later life. For example, anticholinergic medication exposure is a prevalent and potentially modifiable risk factor. Commonly prescribed anticholinergic medications for conditions such as itch or depression could be substituted with alternative therapies with less or no anticholinergic properties, if this change was associated with health benefits.

Previous literature exploring associations between anticholinergic medication exposure and physical function has focused on populations of older adults who are generally >65 years old [4,[8], [9], [10], [11], [12], [13]]. Studies have also been limited by small sample sizes [4,10,12,13] and have used a range of assessment tools to measure anticholinergic medication exposure, some of which have not been well validated [8,12]. For example, a study of 88 community dwelling older adults (mean age 72) measured serum anticholinergic activity and linked this to slower gait speed [13]. A study of 932 moderately to severely disabled community-dwelling older women (>65 years) demonstrated anticholinergic medication exposure, assessed using a novel scale, to be associated with higher odds of slow gait speed, poor balance, poor chair stands performance and weak grip strength [8]. Another study of frail elderly (n = 364, aged >80) used a novel scale to measure anticholinergic medication exposure and linked higher exposure to impaired grip strength and poorer performance in the Short Physical Performance Battery (based on walking speed, balance, and chair stand tests) [12]. A recent case-control study investigating falls on 428 community dwelling older adults (mean age 74) found anticholinergic medication exposure measured with the ACB scale to be significantly associated with poorer dynamic balance and gait speed, but not grip strength, similar to our longitudinal results [4].

Three studies have previously examined the association of Drug Burden Index (DBI) and physical function [[9], [10], [11]]. All three reported DBI to be associated with slower gait speed in older people. However, only one of them reported the effects of the anticholinergic component of the DBI separately. In this study of older Australian men, the anticholinergic part of the DBI was associated with weak grip strength only and not TCSS, usual walking speed or standing balance in contrast to our results [9].

In the prospective analyses, ACB category was demonstrated to predict slower gait speed, standing balance and TCSS performance, but not grip strength. This could indicate that anticholinergic medication exposure affects physical capability not by reducing overall strength, but by acting in the central nervous system (CNS) areas responsible for movement control and balance, something previously suggested by Zia et al. [4]. For example, the Basal Ganglia is a brain area highly involved in movement control and has a high density of cholinergic neurons [26]. Furthermore, most medications with anticholinergic properties are non-selective antagonists of muscarinic acetylcholine receptors [27] and all five muscarinic receptor subtypes are expressed in the brain [28]. Therefore, given the broad distribution of muscarinic receptors in the CNS and the high density of cholinergic neurones in areas important for movement control, such as the basal ganglia, anticholinergic mediations could mediate their effects on physical capability via CNS action.

Our study has several strengths. We used a well validated scale to measure the anticholinergic burden. We also utilised the infrastructure of a large prospective population-based cohort study, which allowed us to capture a sufficient number of participants with high ACB scores to power analyses. The wide age range of the cohort which includes both men and women also improves the generalizability of our results. We were also able to perform both cross-sectional and prospective analyses and control for variety of lifestyle and sociodemographic factors as well as comorbidities.

We also have some limitations. EPIC-Norfolk is a prospective cohort study that was similar to other UK representative population samples at baseline [16]. However, it is possible that attrition bias could have been introduced due to the 10- to 15-year length of the study with only 8623 out of the original 25,000 participants returning for the 3HC [17]. Additionally, we could not control for all potential confounders e.g., prevalent illness was incompletely ascertained at the 3HC. Therefore, some participants could have developed important co-morbidities impacting on their physical capability e.g., arthritis that we did not completely capture at the 3HC because we were relying on information collected at earlier time points (the 1HC and 2HC). Although we were able to calculate the total ACB scale score, we were not able to identify whether particular drugs or dosages were linked to physical capability or whether any associations observed were ubiquitous across all medications with anticholinergic properties. If ubiquitous, this would strengthen evidence that associations were unlikely to be due to a particular underlying health condition for which the medication was prescribed. Finally, associations were stronger and more consistent in women than men in prospective analyses. This could be due to physiological differences between sexes but it should also be noted that there were fewer men in analyses, particularly in the highest ACB category at the 1HC, reducing power.

## 5 Conclusion

The use of anticholinergic medications predicts poor physical capability in the EPIC-Norfolk population. The association between ACB category and objective measures of functional performance remained significant after adjusting for various potential confounders. Our study adds to the growing evidence that suggests clinicians should prescribe drugs with anticholinergic properties with caution. Future studies should explore the effect of reduction in anticholinergic medication prescribing on physical capability.

## Contributors

Kaisa R Yrjana performed literature review and data analysis and interpretation, and drafted the manuscript.

Victoria L Keevil conceived the study, made a significant contribution to the data interpretation, and drafted the manuscript

Roy L Soiza made a significant contribution to the data interpretation and critical appraisal.

Robert N Luben performed data linkage and made a significant contribution to the data interpretation and critical appraisal.

Nicholas J Wareham made a significant contribution to the data interpretation and critical appraisal, and is a PI of the EPIC-Norfolk Cohort.

Kay-Tee Khaw made a significant contribution to the data interpretation and critical appraisal, and is a PI of the EPIC-Norfolk Cohort.

Phyo K Myint conceived the study, and made a significant contribution to the data interpretation and critical appraisal.

All contributed to the writing of the paper. PKM is the guarantor.

## Conflict of interest

The authors, Kaisa R Yrjana, Victoria L Keevil, Roy L Soiza, Robert N Luben, Nicholas J Wareham, Kay-Tee Khaw and Phyo K Myint, have no conflicts of interest that are directly relevant to the content of this work. However, the following is declared.

## Funding

The EPIC-Norfolk study has been supported by grants from 10.13039/501100007155Medical Research Council [grant numbers G9502233, G0401527] and 10.13039/501100000289Cancer Research UK [grant number C864/A8257]; and the third health examination clinic was supported by a grant from Research into Ageing [grant number 262]. The funders have had no roles in the study design or interpretation of the results. VLK is supported by a MRC/NIHR Clinical Academic Research Partnership Grant. KRY is currently employed as a regulatory affairs student assistant at Lundbeck (Ottiliavej 9, 2500 Copenhagen, Denmark).

## Ethics

Signed informed consent was obtained from the participants at baseline and renewed at the third health examination. Ethical approval was obtained from the Norfolk Local Research Ethics Committee and the East Norfolk and Waveney NHS Research Governance Committee.

## Data sharing and collaboration

There are no linked research data sets for this paper. Data from the EPIC-Norfolk study is confidential and only shared with study collaborators. Collaboration and data requests can be made to the Data Management Committee.

## Provenance and peer review

This article was not commissioned and was externally peer reviewed.
